# High type I error and misrepresentations in search for transgenerational epigenetic inheritance: response to Guerrero-Bosagna

**DOI:** 10.1186/s13059-016-0981-5

**Published:** 2016-07-12

**Authors:** Khursheed Iqbal, Diana A. Tran, Arthur X. Li, Charles Warden, Angela Y. Bai, Purnima Singh, Zach B. Madaj, Mary E. Winn, Xiwei Wu, Gerd P. Pfeifer, Piroska E. Szabó

**Affiliations:** Beckman Research Institute Irell and Manella Graduate School of Biological Sciences, Duarte, CA USA; Eugene and Ruth Roberts Summer Academy, Duarte, CA USA; Van Andel Research Institute, Center for Epigenetics, 333 Bostwick Ave, Grand Rapids, MI 49503 USA

## Abstract

In a recent paper, we described our efforts in search for evidence supporting epigenetic transgenerational inheritance caused by endocrine disrupter chemicals. One aspect of our study was to compare genome-wide DNA methylation changes in the vinclozolin-exposed fetal male germ cells (n = 3) to control samples (n = 3), their counterparts in the next, unexposed, generation (n = 3 + 3) and also in adult spermatozoa (n = 2 + 2) in both generations. We reported finding zero common hits in the intersection of these four comparisons. In our interpretation, this result did not support the notion that DNA methylation provides a mechanism for a vinclozolin-induced transgenerational male infertility phenotype. In response to criticism by Guerrero-Bosagna regarding our statistical power in the above study, here we provide power calculations to clarify the statistical power of our study and to show the validity of our conclusions. We also explain here how our data is misinterpreted in the commentary by Guerrero-Bosagna by leaving out important data points from consideration.

Please see related Correspondence article: xxx (13059_2016_982) and related Research article: http://genomebiology.biomedcentral.com/articles/10.1186/s13059-015-0619-z

Here we have reassessed the statistical power of our study [1] where we compared genome-wide DNA methylation changes in the in utero-exposed, reprogrammed (G1R) fetal male germ cells (MGC) to control samples (n = 3 treated vs. n = 3 control). In the same study we also assessed DNA methylation changes in MGC of the next, unexposed, generation (G2R) (n = 3 vs. n = 3) and also in adult spermatozoa (n = 2 vs. n = 2) in both generations. Of the several factors that determine the statistical power of a study, effect size can have one of the largest impacts. For t-tests, effect size is dependent on the variability of the populations and the precision of the measurements. Here (Table 1) we provide the actual empirical standard deviation, effect, power, and required sample size values for the G1R MGC (n = 3) and G1R sperm (n = 2) comparisons (comparison numbers are as in [1]). As can be seen from Table 1, we cannot reproduce Guerrero-Bosagna’s power calculations, likely because those were not based on our primary data. We had an average of about 0.9998 statistical power to detect a twofold change and 0.927 power to detect a 1.5-fold change using MGC samples (n = 3 vs. n = 3) per group. It is less relevant for the overall interpretation (see the argument below), but we had lower statistical power (0.89 and 0.88 power to detect a twofold change and 0.55 and 0.53 power to detect a 1.5-fold change in G1R sperm (n = 2 vs. n = 2) oil-VZ and VZ-oil comparison (Table 1).

**Table 1:** Power calculations based on primary data in [1]. Unadjusted power and total sample size calculations were done in R v3.2.2 (via the pwr.t.test function in the pwr package [7]. Effect sizes were Cohen’s d. The difference in means was the corresponding log2 fold change. SD was estimated as the pooled standard deviation for a given comparison. These power calculations did not assume false discovery rate (FDR) adjustments because we were concerned that it would be overly conservative and remove potential true positives (tests were not necessarily independent). Further, the inclusion of the false positives that the FDR corrections would have removed should actually increase our odds of identifying any transgenerational effects (whether they were true or false positives), but even under these more relaxed conditions, none could be established. FDR corrected calculations are also displayed in the last two columns. These were done in R via the ssize.twoSamp function from the ssize.fdr package [8]. A true positive ratio of 0.05 was assumed for the FDR calculations

Comparison number	Sample size (n treated + n control)	Pooled AVG STDV	Power for log(2) change	Power to detect 50 % change	Effect size for 50 % difference power calculation	Number of samples to detect 1.5-fold change with power = 0.9	Number of samples to detect 1.5-fold change with power = 0.8	Number of samples to detect 1.2-fold change with power = 0.9	Number of samples to detect 1.2-fold change with power = 0.8	Number of samples to have 5 % FDR in detecting 1.2-fold change	Number of samples to have 5 % FDR in detecting 1.05-fold change
1	6	0.16	1.00	0.92	3.75	6	6	18	14	32	370
2	6	0.16	1.00	0.91	3.64	6	6	18	14	34	392
3	6	0.15	1.00	0.94	3.96	6	6	16	14	30	332
4	6	0.16	1.00	0.90	3.57	8	6	20	16	34	408
5	6	0.15	1.00	0.93	3.80	6	6	18	14	32	362
6	6	0.16	1.00	0.90	3.56	8	6	20	16	34	410
13	4	0.15	0.89	0.55	3.93	6	6	16	14	30	338
14	4	0.15	0.88	0.53	3.78	6	6	18	14	32	364
17	6	0.15	1.00	0.93	3.83	6	6	18	14	30	354
18	6	0.16	1.00	0.92	3.71	6	6	18	14	32	378
19	6	0.14	1.00	0.97	4.26	6	6	14	12	26	288
20	6	0.15	1.00	0.94	3.90	6	6	16	14	30	342
21	6	0.15	1.00	0.95	4.02	6	6	16	12	28	322
22	6	0.15	1.00	0.93	3.84	6	6	18	14	30	352
29	4	0.15	0.89	0.55	3.91	6	6	16	14	30	340
30	4	0.16	0.85	0.49	3.57	8	6	20	16	34	406

As we showed in Table 1, we were very well powered to detect twofold or 1.5-fold changes of DNA methylation in the genome in MGC using sample number n = 3 treated vs. n = 3 control. We detected two hits among vinclozolin (VZ) G1R MGC samples that represented greater than 1.5-fold change (with *P* <0.05), but these were not present in the similarly powered G2R MGC comparisons (n = 3 vs. n = 3). When depicting the results from our Table three [1], in his Fig. 1, Guerrero-Bosagna has left out two important comparisons, OIL-VZ and VZ-OIL methylation changes between G1R and G2R MGC. These missing comparisons are now shown here in Fig. 1. There were zero hits in the G1R-G2R intersections, giving no support in finding a DNA methylation aberration inherited from exposed G1R MGC to unexposed G2R MGC. Consequently, even if we increased our sample size from n = 2 to n = 3 in the sperm comparisons and detected additional significant (greater than 1.5-fold, *P* <0.05) changes in G1R sperm and G2R sperm, those could not be considered pure TGEI changes that originated in G1R MGC and maintained into G2R MGC (Fig. 2).

**Fig. 1:**
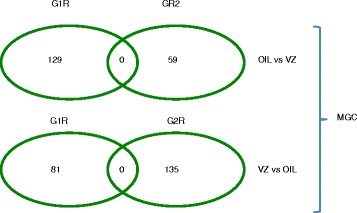
The missing balloons from Fig. 1 of Guerrero-Bosagna’s commentary. Level 2 analyses A, B, E, F, G, and H were depicted as *balloons* from our Table three of [1] in Fig. 1 of the commentary. Analyses C and D were left out and are depicted here: the *intersections* of oil-VZ and VZ-oil comparisons in G1R vs. G2R generations. Note that the intersection in both cases contains zero hits. Note also that we had near 1.0 statistical power to detect a twofold change and 0.94 and 0.9 power to detect a 50 % change in these comparisons (see Table 1)

**Fig. 2:**
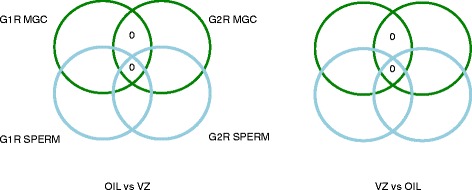
Interpretation of our data explained. Level four comparisons are depicted from Table three of [1], showing zero numbers in two critical intersections. When interpreting our data, we considered a methylation change between experimental and control samples to be true transgenerational epigenetic inheritance (TGEI) if we found it as a common hit in four comparisons involving G1R MGC, G1R sperm, G2R MGC, and G2R sperm. Finding zero hits in the intersection of four comparisons was interpreted as lack of evidence for TGEI. For example, finding a common hit between G1R MGC and G1R sperm but not in G2R samples meant that the aberration was erased in G2. Similarly, finding it in G2R but not in G1R samples meant that it did not originate from the G1 exposure. Finding it in G1R and G2R MGC but not in sperm samples meant that it could not be transmitted between generations by G1R sperm or G2R sperm. Note that level 2 and 3 analyses were shown as balloons but level four analyses were omitted from the commentary by Guerrero-Bosagna

The statistical power in our study was not sufficient to detect small methylation changes (i.e. 1.2-fold), but we do not believe these are biologically significant differences. It is highly unlikely that such small aberrations cause the robust male infertility phenotype described by Anway et al. that occurs with high penetrance to “nearly all males of subsequent generations examined (that is, F1 to F4)” and “the effects on reproduction correlate with altered DNA methylation patterns in the germ line” [2].

It is also interesting to note that given our standard deviation values we would need to use a total of 122–174 samples to detect a 1.05-fold change with 0.8 power or 288–408 samples to avoid false-positive changes at the 1.05-fold change level. This latter is about the number Guerrero-Bosagna would have needed to avoid type I error in his study [3], assuming that he had similar precision/reproducibility measures to our experiment. Unfortunately, he only used two samples (even though he pooled sperm from three individuals into each of the two samples). Pooling three samples into two replicates may provide statistical power beyond n = 2 v. n = 2 comparisons based on extrapolating from studies that use computational simulations with larger numbers [4, 5]. Those simulations involve many assumptions and still have to be validated experimentally and when using small n-s such that was used by Guerrero-Bosagna. He did not use any fold-change cutoff values in his relevant study involving F3 sperm of VZ-treated rats. In that study, he reported even a 0.7 % change as significant and confirmed at the *Eef1d* promoter between VZ and control samples [3], see Table one and Fig. two in reference [3].

Our study was well-powered in G1R and G2R prospermatogonia to detect the top hits (20-fold and 0.2-fold change by real-time PCR depicted in Fig. four of the mouse study by Guerrero-Bosagna [6]), but we have failed to confirm those (see Fig. S nine in our paper [1]). Two replicate MIRA-chip samples out of three G1R and G2R samples are displayed in our Figs. seven, eight, and S nine and show the level of reproducibility in our hands [1]. Guerrero-Bosagna did not display any duplicate measurements in his MeDiP studies, so it is not possible to get a visual assurance for the level of reproducibility. He was unable to validate by independent methods a larger number of hits than the number he validated [3, 6], further suggesting random effects. There was no overlap in DNA methylation hits between his rat and mouse experiments, likely because of the large type-I error in those studies. The link still has to be shown between G3 sperm methylation and the primary aberration in the exposed germ cells.

With our statistical power to detect what we consider to be biologically significant differences in DNA methylation, we cannot provide evidence for TGEI by VZ (and the other endocrine disrupter chemicals) treatment. Clearly, further well-designed, carefully executed, and statistically well-powered studies are needed for evaluating TGEI in mammals. Indeed, studies that support TGEI should also be scrutinized for statistical stringency. At the end, the functional role of any putative TGEI will need to be genetically validated.
